# Role of ferroptosis on tumor progression and immunotherapy

**DOI:** 10.1038/s41420-022-01218-8

**Published:** 2022-10-26

**Authors:** Deting Gong, Mingjun Chen, Yuhan Wang, Juanjuan Shi, Yongzhong Hou

**Affiliations:** School of Life Sciences, Jiangsu University, Zhenjiang, Jiangsu 212013 PR China

**Keywords:** Immunosurveillance, Cell death, Ubiquitin ligases

## Abstract

Ferroptosis is triggered by intracellular iron leading to accumulation of lipid peroxidation consequent promotion of cell death. Cancer cell exhibits ability to evade ferroptosis by activation of antioxidant signaling pathways such as SLC7A11/GPX4 axis. In addition to transcriptional regulation on ferroptosis by NRF2, SREBP1, YAP, and p53, ferroptosis is modulated by ubiquitination or autophagic degradation. Moreover, zinc or Ca^2+^ could modulate ferroptosis by inducing lipid peroxidation and ferroptosis. Induction of ferroptosis enhances immune cell activity such as T cells or macrophages, which is associated with the release of DAMPs (damage-associated molecular patterns) and IFNγ. Therefore, combined immune checkpoint inhibitors with ferroptosis inducers effectively enhance antitumor immunotherapy, whereas induction of ferroptosis could impair T cell activity or survival, suggesting that rational combined therapy for cancer is essential. In this review, we discussed the regulatory role of ferroptosis on tumor progression and immunotherapy.

## Facts

1. Ferroptosis is one kind of cell death except apoptosis, necrosis, and autophagy.
2. In addition to transcriptional regulation, ferroptosis is modulated by ubiquitination or autophagic degradation.
3. Ferroptosis is associated with antitumor immunotherapy.

## Open questions

1. Do other GPX4 family members including GPX1-3 regulate ferroptosis?
2. Is there any other autophagy receptor for ferritin degradation except NCOA4?
3. Does GPX4 undergo ubiquitination and degradation?
4. Does SLC7A11 undergo autophagic degradation?
5. What is the mechanism of zinc and Ca^2+^ on ferroptosis?

## Introduction

**Fig. 1:**
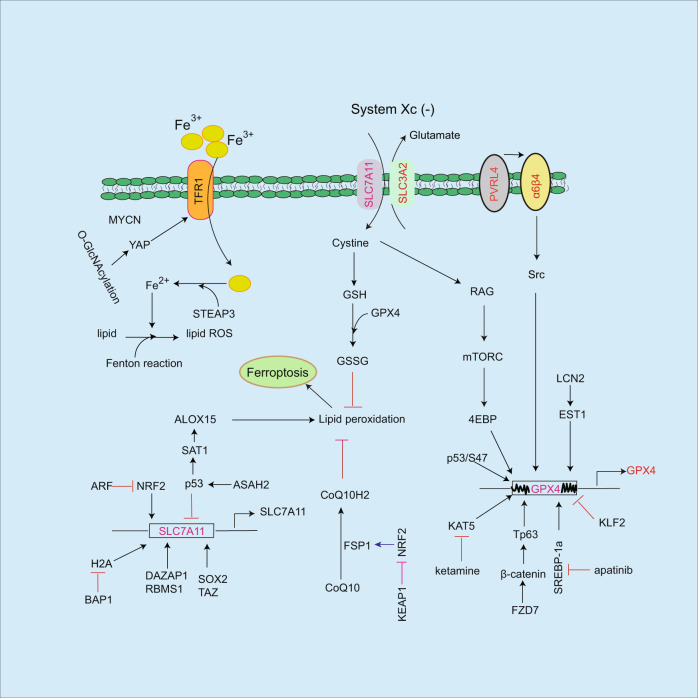
Regulating pathways of ferroptosis. TFR1 triggers iron uptake, which is regulated by MYCN and YAP pathways. Accumulation of iron facilitates lipid peroxidation and ferroptosis. In contrast, SLC7A11/GPX4 axis prevents ferroptosis by antioxidant role. SLC7A11 or GPX4 expressions are regulated by multiple regulators including NRF2, SOX2, TAZ, EST1, and SREBP-1a etc. Moreover, SLC7A11 mediates cystine uptake, subsequently, activates Rag-mTORC1-4EBP pathway-induced GPX4 protein synthesis. Ferroptosis resistance is blocked by some mediators such as p53, KLF2 etc. In addition to GPX4, FSP1 is a ferroptosis inhibitor by inhibiting lipid ROS accumulation, which is reversed by KEAP1/NRF2 signaling pathway.

**Fig. 2:**
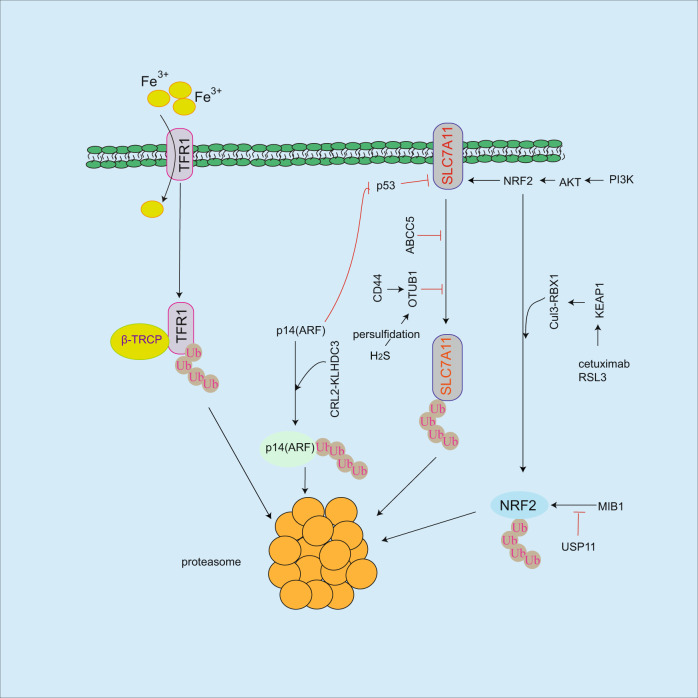
Regulation of ubiquitination on ferroptosis. Although TFR1 facilitates iron uptake, it will be degraded by β-TRCP ubiquitin ligase leading to ferroptosis resistance. OTUB1 or ABCC5 inhibits SLC7A11 ubiquitination and degradation, and CD44 or H_2_S-mediated persulfidation of OTUB1 enhances this event. However, it is unclear the mechanism of SLC7A11 degradation in proteasome. MIB1 ubiquitin ligase induces NRF2 ubiquitination and degradation, which is reversed by USP11 deubiquitinating enzyme. Under base condition, the interaction of KEAP1 with NRF2 recruits Cul3-RBX1 ubiquitin ligase to NRF2 for proteasomal-dependent degradation in response to ferroptosis inducers (RSL3, cetuximab). However, in response to oxidative stress, KEAP1 dissociates from NRF2 and increases NRF2 protein stability resulting in ferroptosis resistance. Although p14(ARF)/p53 pathway inhibits SLC7A11 resulting in ferroptosis sensitivity, p14(ARF) is degraded by CRL2-KLHDC3 ubiquitin ligase, which in turn causes ferroptosis resistance.

**Fig. 3:**
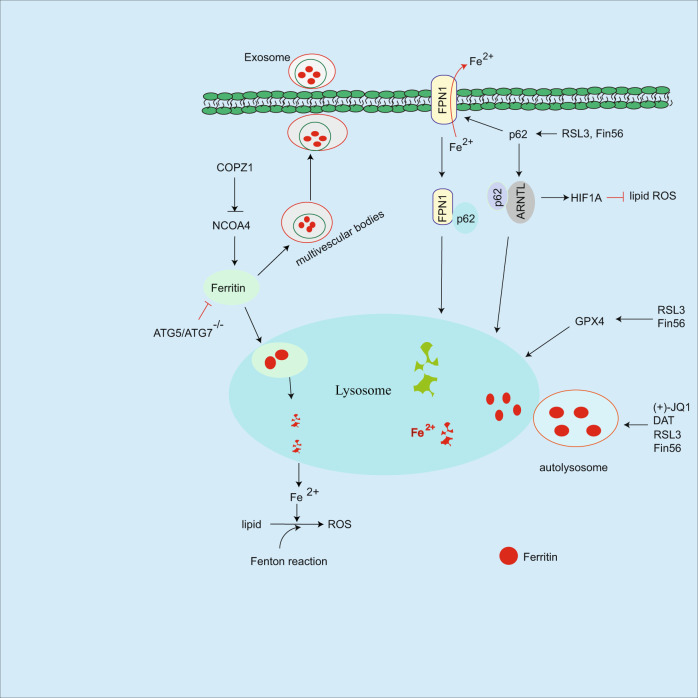
Regulation of autophagy on ferroptosis. NCOA4 induces ferritin autophagic degradation leading to the release of iron, which in turn facilitates ferroptosis, while COPZ1 inhibits NCOA4/ferritin pathway. Ferritin could be embedded into multivesicular bodies and traffic out of cells as exosome leading to reduced intracellular iron levels and ferroptosis resistance. In response to inducers, p62-mediated FPN1 or ARNTL autophagic degradation, which in turn promote sensitivity of ferroptosis. Although ferritinophagy is triggered in response to inducers including RSL3, Fin56, DAT etc, the mechanism is unclear.

**Fig. 4:**
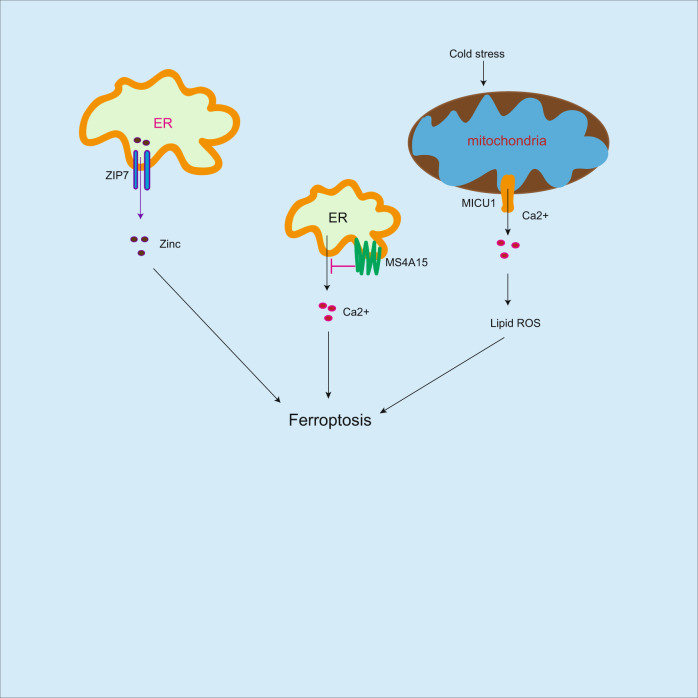
Regulation of ferroptosis by metallic irons. In addition to iron, zinc and calcium could induce ferroptosis. ZIP7 mediates zinc release from ER to cytosol resulting in ferroptosis. In addition, cold stress increases lipid peroxidation accumulation and induces ferroptosis, which is associated with MICU1-dependent mitochondrial Ca2+ uptake. However, calcium (Ca2+)-mediated ferroptosis could be blocked by MS4A15, which in turn inhibits ferroptosis.

**Fig. 5:**
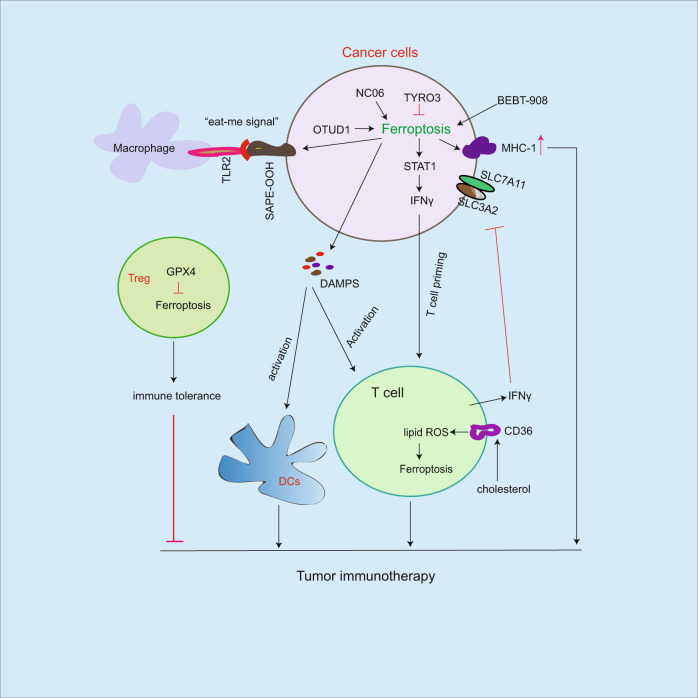
Role of ferroptosis on cancer immunotherapy. Ferroptosis induction facilitates MCH-1 expression, release of DAMPS and IFNγ, which in turn activate immune cell activity including T cells and macrophages. In addition, the release of IFNγ from T cells inhibits Xc (-) system (SLC7A11/SLC3A2), leading to increased ferroptosis sensitivity. Ferroptosis resistance by GPX4 in Treg cells causes immune tolerance. As an eat-me signal, SAPE-OOH is distributed on ferroptotic cancer cells, which is recognized by TLR2 on macrophages leading to increased phagocytosis. Although combined immune checkpoint inhibitors with ferroptosis inducers could effectively enhance antitumor immunotherapy, induction of ferroptosis could impair T cell survival by ferroptosis. Therefore, a rational combined therapy is essential.

Ferroptosis is one kind of cell death except apoptosis, necrosis, and autophagy, which is iron-dependent manner and was named by Dixon in 2012 [1]. Erastin is the first reagent to induce cell ferroptosis [2], which is also the inhibitor of system Xc (-). As acystine/glutamate antiporter, Xc(-) plays an important role in preventing ferroptosis by cystine uptake and glutamate export, and activation of GPX4 (glutathione peroxidase 4) mediates antioxidant process leading to ferroptosis resistance [3–5]. As one of the important components in system Xc (-), SLC7A11 (solute carrier family 7 member 11) expression is regulated by multiple signaling pathways including NRF2 (nuclear factor erythroid 2-related factor 2) [6], SOX2 (sry-box transcription factor-2) [7], yes-associated protein (YAP)/tafazzin (TAZ) [8], and ABCC5 (ATP binding cassette subfamily C member 5) [9]. SLC7A11/GPX4 axis exhibits antioxidant role by reducing lipid peroxidation accumulation resulting in the inhibition of ferroptosis [10], whereas blockade of SLC7A11 or GPX4 promotes cell ferroptosis [5]. In addition to SLC7A11, SLC2A1 (solute carrier family 2 member 1), also known as Glut1 (glucose transporter 1), promotes glucose uptake and fatty acid synthesis, consequently facilitating lipid peroxidation-dependent ferroptosis [11]. Polyunsaturated fatty acid biosynthesis modulates gastric cancer cell ferroptosis [12], which is blocked by α6β4/SRC/STAT3-mediated inhibition of ACSL4 (acyl-coA synthetase long-chain family member 4) [13]. HIF-2α increases polyunsaturated lipids [14], and iron regulatory gene expressions, which in turn facilitates ferroptosis [15], but VHL (von hippel-lindau syndrome) mediates HIF-2α degradation leading to ferroptosis resistance [16]. As iron-dependent cell death, iron uptake is regulated by TFR1 (transferrin receptor 1) that acts as a critical role in triggering ferroptosis [17]. Ferroptosis not only inhibits tumor growth but also enhances cancer cell immunotherapy by multiple signaling pathways [18–20] (Figs. 1–5). In this review, we discussed the regulatory role of ferroptosis on cancer progression and immunotherapy.

## Regulating pathways of ferroptosis

SLC7A11 is essential for inhibition of ferroptosis via cystine uptake leading to activation of downstream GPX4, subsequently, blocks lipid peroxidation accumulation and inhibits ferroptosis. Cancer cells exhibit the ability to prevent ferroptosis by expressing high levels of SLC7A11, which is regulated by NRF2 [6], SOX2 in lung cancer stem-like cells [7], and YAP/TAZ pathway in hepatocellular carcinoma (HCC) [8] leading to ferroptosis resistance (Fig. 1). As an inhibitor of ferroptosis, DAZ associated protein 1 (DAZAP1) directly binds to SLC7A11 mRNA leading to increased SLC7A11 transcription activity, which in turn inhibits ferroptosis in response to sorafenib in HCC [21]. Similarly, RNA binding motif single-stranded interacting protein 1 (RBMS1)/ElF3d (eukaryotic initiation factor 3d) pathway increases SLC7A11 gene transcription [22]. However, BRCA1-associated protein 1 (BAP1) blocks ubiquitination of histone 2A leading to inhibition of SLC7A11 gene transcription, subsequently, increases lipid peroxidation and promotes cancer cell ferroptosis [23]. In recurrent breast tumors, epithelial-mesenchymal transition-induced discoidin domain receptor 2 expression facilitates breast cancer cell growth, while it increases the sensitivity of ferroptosis by activation of YAP/TAZ pathway in response to erastin [24]. However, YAP/TAZ pathway-mediated SLC7A11 expression inhibits ferroptosis in HCC [8]. This contradictory observation may be derived from different experimental contexts.

SLC7A11 facilitates cystine uptake consequent activation of downstream GPX4 pathway, subsequently, prevents lipid peroxidation accumulation and inhibits ferroptosis [10, 25]. GPX4 is upregulated by lipocalin-2 in colorectal cancer [26], SREBP-1a in gastric cancer cells [27], KAT5 in breast cancer cells [4], FZD7-β-catenin-Tp63 pathway in ovarian cancer cells [28] and PVRL4/α6β4/Src pathway [29] (Fig. 1). Conversely, inhibition of GPX4 by apatinib [27], ketamine [4], and HDL-like nanoparticles [30] facilitates ferroptosis. kruppellike factor 2 directly inhibits GPX4 gene transcription, consequently reduces GPX4 levels and promotes ferroptosis in clear cell renal cell carcinoma [31]. In addition, isocitrate dehydrogenase 1 mutation reduces GPX4 protein levels and induces ferroptosis in response to erastin in HT-1080 cells [32]. In addition to regulation of GPX4 gene expression, GPX4 protein undergoes degradation [33]. Activating transcription factor 4 mediates heat shock protein family A (Hsp70) member 5 expression, which in turn binds to GPX4 resulting in increased GPX4 protein stability with unclear mechanism in pancreatic ductal adenocarcinoma cells [33]. The link of SLC7A11 to GPX4 is further determined [34], SLC7A11 mediates cystine uptake, subsequently, activates Rag-mTORC1–4EBP pathway-induced GPX4 protein synthesis. Conversely, inhibition of mTORC1 decreases GPX4 expression and facilitates ferroptosis sensitivity in cancer cells. These findings suggest that SLC7A11 not only promotes GPX4-mediated detoxification but also increases GPX4 expression by cystine/mTOR/4EBP1 pathway (Fig. 1).

As a transcription factor, NRF2 blocks ferroptosis by upregulation of multiple gene expressions including SLC7A11 [6], heme oxygenase-1 (HO-1) [35], metallothionein-1G (MT-1G) [36] and glutamate-cysteine ligase catalytic subunit (GCLC) [37], consequently, inhibits lipid peroxidation and ferroptosis (Fig. 1). In response to sorafenib, NRF2 induces MT-1G expression in HCC leading to inhibition of ferroptosis, while silence of MT-1G reverses this event [36]. Although cystine starvation increases ferroptosis sensitivity, it could induce NRF2-mediated GCLC expression leading to gamma-glutamyl-peptides accumulation, which in turn inhibits glutamate accumulation and ferroptosis in NCSC [37], suggesting that induction of ferroptosis in some contexts could cause negative feedback regulation in cancer cells. NRF2 can induce MRP1 (multidrug resistance protein 1) expression, which is resistant to cytotoxic drugs [38], while MRP1 mediates intracellular glutathione exflux leading to increased ferroptosis sensitivity in HAP1 cells in response to erastin 2, suggesting that high NRF2 level does not limit ferroptosis in response to inducers [39]. Although NRF2 induces SLC7A11 gene expression and inhibits ferroptosis, ADP-ribosylation factor (ARF) blocks this event independent of p53 [6]. As a tumor suppressor, p53 directly inhibits SLC7A11 gene transcription and promotes ferroptosis [40–42]. As a direct target of p53, activation of spermidine/spermine N1-acetyltransferase 1 accumulates lipid peroxidation and sensitizes to ferroptosis, which is associated with p53/SAT1-mediated arachidonate 15-lipoxygenase expression [43]. In response to radiotherapy, activation of p53 inhibits SLC7A11 expression resulting in ferroptosis in response to inducers [42], suggesting that combined ferroptosis inducers could effectively enhance cancer therapy. However, p53-mediated ferroptosis is abolished by N-acylsphingosine amidohydrolase 2 (ASAH2)-induced p53 degradation leading to inhibition of ferroptosis in myeloid-derived suppressor cells (MDSCs) [44], whereas the mechanism of p53 degradation is unclear. In addition to wild type, p53 mutation p53(3KR) has no effect on cancer cell cycle arrest, apoptosis, and senescence, while it still induces ferroptosis by inhibiting SLC7A11 expression in response to oxidative stimuli [41]. However, S47 variant of p53 is resistant to ferroptosis by increasing GPX4 levels [45]. In contrast to increased ferroptosis sensitivity, p53 limits ferroptosis by inhibiting the activity of DPP4 (dipeptidyl-peptidase-4) in colorectal cancer, which is involved in blockade of DPP4-dependent lipid peroxidation [46]. In response to cystine deprivation, p53-mediated p21 expression reduces both of cellular glutathione and ROS levels leading to the inhibition of ferroptosis [47]. These findings suggest that p53 exhibits dual role in regulation of ferroptosis (Fig. 1).

In addition to GPX4, ferroptosis suppressor protein 1 (FSP1) is another ferroptosis inhibitor by inducing ubiquinol (CoQ10H2) generation from ubiquinone (CoQ10), and then FSP1-CoQ10-NAP(p)H pathway reduces lipid peroxidation accumulation resulting in inhibition of cancer cell ferroptosis [48, 49]. However, KEAP1/NRF2 signaling pathway increases lung cancer cell ferroptosis by inhibiting FSP1 expression [50] (Fig. 1).

Ferroptosis is a type of non-programmed death, which is iron-dependent cell death, therefore intracellular iron levels are critical for ferroptosis. In this process, TFR1 plays an important role in promoting iron uptake. MYCN induces TFR1 expression in neuroblastoma cancer cells resulting in increased iron levels and lipid ROS production, consequently facilitates ferroptosis in response to inducers such as SAS and auranofin [17]. In addition, YAP increases TFR1 expression resulting in accumulation of intracellular iron levels, and O-GlcNAcylation of YAP enhances this event in HCC [51]. Conversely, intracellular iron levels could be reduced in sterol regulatory element binding protein-2 (SREBP2) expressed circulating melanoma cells from patients. Mechanistically, SREBP2 induces the expression of transferrin resulting in blockade of accumulation of intracellular iron, consequently causes ferroptosis resistance in response to inducers [52]. These findings suggest that TFR1 exhibits an important role in regulating intracellular iron levels and ferroptosis (Fig. 2).

## Regulation of ferroptosis by ubiquitination

Ubiquitin-proteasome system acts as an important role in regulation of protein stability [53–56]. In ferroptosis process, several regulators undergo ubiquitination and degradation including SLC7A11 [57, 58], NRF2 [59, 60], and p14(ARF) [61] (Fig. 2). As a deubiquitinating enzyme, OTUB1 increases SLC7A11 protein stability by blocking its ubiquitination [57, 58]. The binding of OTUB1 to SLC7A11 leads to inhibition of SLC7A11 degradation. Moreover, CD44, a cancer stem cell marker, enhances this event by increasing the interaction of SLC7A11 with OTUB1 [57]. OTUB1 persulfidation modification by intracellular hydrogen sulfide (H_2_S) enhances SLC7A11 stability, consequently inhibits colon cancer cell ferroptosis [58]. These findings suggest that SLC7A11 undergoes ubiquitination and degradation, which is blocked by OTUB1. However, it is still unclear the degrading mechanism of SLC7A11 by proteasome. Furthermore, although NRF2 inhibits ferroptosis by upregulation of multiple gene expressions including SLC7A11 [6], HO-1 [35], MT-1G [36], and GCLC [37], NRF2 undergoes ubiquitination and degradation by KEAP1/Cul3-RBX1 E3 ubiquitin ligase complex [59, 60]. Under base condition, the binding of KEAP1 to NRF2 recruits Cul3-RBX1 E3 ubiquitin ligase to NRF2 for ubiquitination and degradation. Conversely, in response to oxidative stress, KEAP1 is dissociated from NRF2 resulting in increased NRF2 protein stability [59, 60], suggesting that KEAP1 regulates NRF2 protein stability. NRF2-mediates HO-1 expression and ferroptosis resistance in KRAS mutant colorectal cancer cells, whereas combined cetuximab with RSL3 increases KEAP1 expression leading to inhibition of NRF2/HO-1 pathway [35]. In addition, MIB1 ubiquitin ligase facilitates NRF2 degradation resulting in ferroptosis in response to inducers [62], but USP11 deubiquitinating enzyme reverses this process leading to increased NRF2 protein stability in non-small cell lung cancer (NSCLC) [63]. These findings suggest that NRF2-mediated ferroptosis resistances by regulating multiple gene expressions, while its protein will be degraded by ubiquitination leading to increased ferroptosis sensitivity. p14(ARF) induces ferroptosis by inhibition of NRF2-mediated SLC7A11 expression, while CRL2-KLHDC3 E3 ubiquitin ligase complex induces p14(ARF) proteasomal-dependent degradation leading to ferroptosis resistance [61]. As an important regulator of iron uptake, TFR1 undergoes ubiquitination and degradation by β-TRCP E3 ligase in a tribbles pseudokinase 2 (TRIB2)-dependent manner, consequently blocks ferroptosis in live cancer cells [64], but how does TRIB2 affect βTRCP-mediated TFR1 degradation? which needs to be further identified.

## Regulation of ferroptosis by autophagy

Autophagy regulates cancer progression and immune response by degrading cellular components including proteins, mitochondria etc. [18, 19, 65]. As an iron storage protein, intracellular ferritin undergoes autophagic degradation in lysosome leading to release of iron, subsequently, increased intracellular iron levels facilitate ferroptosis sensitivity. Conversely, autophagy deficiency in Atg5 or Atg7 silenced cancer cells abolishes this event [66]. In this process, nuclear receptor coactivator 4 (NCOA4) acts as a selective autophagy receptor to mediate ferritin lysosomal-dependent degradation, which in turn promotes release of iron, also known as ferritinophagy [67]. In contrast, COPZ1 (coatomer protein complex subunit zeta 1) inhibits NCOA4 expression, while silenced COPZ1 increases NCOA4 protein levels and promotes ferritinophagy in glioblastoma multiforme [68]. In addition to NCOA4, autophagy receptor SQSTM1/p62 mediates iron exporter FPN1 (ferroportin1) degradation in lysosome, subsequently accumulates cellular iron levels, and facilitates ferroptosis [69]. In response to RSL3 or Fin56, p62-mediated aryl hydrocarbon receptor nuclear translocator-like (ARNTL) autophagic degradation, consequently inhibits HIF1A-mediated ferroptosis resistance [70, 71]. RSL3 can block mTOR activation leading to autophagy induction and GPX4 autophagic degradation in human pancreatic cancer cells [72]. In addition, Fin56 can also induce GPX4 autophagic degradation and combined Fin56 with mTOR inhibitor enhances bladder cancer cell ferroptosis [73], suggesting that the combination of autophagy and ferroptosis inducers could effectively enhance cancer therapy. GOT1 inhibition increases intracellular iron levels, which is associated with increased ferritinophagy in pancreatic cancer cells, while the mechanism is unclear [74]. In addition, ferritinophagy is induced in response to (+)-JQ1 [75], supraphysiologic testosterone in prostate cancer [76], and dihydroartemisinin [77], or some ferroptosis inducers RSL3, Fin 56, and zalcitabine [70, 71, 78, 79], suggesting that autophagy modulates intracellular iron level by lysosomal degradation of ferritin (Fig. 3). However, how do these ferroptosis inducers trigger ferritin autophagic degradation? This issue needs to be further determined.

## Regulation of ferroptosis by metallic ions

In addition to iron, zinc can induce ferroptosis [80]. Zinc increases breast and renal cancer cell sensitivity to ferroptosis. Mechanistically, zinc transporter-7 (ZIP7) mediates zinc release from endoplasmic reticulum to cytosol, which in turn enhances ferroptosis. Cold stress increases lipid peroxidation accumulation and induces ferroptosis in kidney- and liver-derived cell lines, which is associated with (mitochondrial calcium uptake 1-dependent mitochondrial Ca^2+^ uptake [81]. However, calcium (Ca^2+^)-mediated ferroptosis could be suppressed by membrane-spanning 4-domains A15, the endoplasmic reticulum protein, which depletes luminal Ca^2+^ stores leading to inhibition of lipid peroxidation accumulation and ferroptosis [82]. Ferroptosis is iron-dependent cell death, while zinc and Ca^2+^ [80–82], can induce lipid peroxidation and ferroptosis (Fig. 4), which expand the conception of iron-dependent death, but the mechanism needs to be further identified.

## Regulation of ferroptosis on tumor immunotherapy and radiotherapy

Cancer cell exhibits ability to evade immunotherapy by regulating immune checkpoint signaling pathways, subsequently, escape immune cell surveillance including T cells, macrophages, and dendric cells [18–20]. Induction of ferroptosis increases CD8(+) T cell activity [44] and enhances tumor immunotherapy [83]. NC06 treatment triggers ferroptosis in MDSC by inhibiting ASAH2 and enhances CD8(+) T cell activity in tumors [44]. In contrast, TYRO3-mediated ferroptosis resistance leads to reduced anti-PD-1/PD-L1 antitumor immunotherapy [83]. MCH-1 undergoes autophagic degradation in pancreatic ductal adenocarcinoma cells leading to escape of immune surveillance [84]. BEBT-908, a dual PI3K/HDAC inhibitor, induces ferroptosis resulting in increased MCH-1 expression and activation of STAT1/IFNγ signaling in cancer cells, which in turn promotes antitumor immunotherapy [85]. In tumor microenvironment, the released IFNγ from CD8(+) T cells suppresses SLC3A2 and SLC7A11 expression on cancer cells, consequently facilitates cancer cell accumulation of lipid peroxidation and ferroptosis [86]. In response to radiotherapy, the released IFNγ from CD8(+) T cells synergistically inhibits SLC7A11 expression leading to activation of ferroptosis, which in turn enhances anti-PD-L1 antitumor immunotherapy [87]. These findings suggest that the additional role of T cell killing to cancer cells by induction of ferroptosis. Since cyst(e)inase treatment can induce intracellular cystine degradation consequent promotion of cancer cell ferroptosis [88], the combined cyst(e)inase with PD-L1 antibody effectively enhances tumor immunotherapy [86], suggesting that combined ferroptosis inducers with immune checkpoint inhibitors could effectively enhance cancer immunotherapy. In contrast to T cell killing to cancer cells by ferroptosis induction, in tumor microenvironment, cholesterol-mediated CD8(+) T cell ferroptosis leads to cancer cell immune escape [89]. In this process, cholesterol induces CD36 expression on tumor-infiltrating CD8(+) T cells, consequently, CD36 triggers uptake of fatty acids in CD8(+) T cells resulting in ferroptosis. In contrast, block of CD36 enhancesant-PD-1 antitumor immunotherapy [89], suggesting that ferroptosis inducers could impair T cell survival. In contrast to CD8(+) T cell killing to cancer cells, T regulatory (Treg) cells exhibit immune tolerance and inhibit antitumor immunotherapy, and high levels of GPX4 in Treg cells prevent lipid peroxidation and ferroptosis. In contrast, blockade of GPX4 enhances antitumor immunotherapy [90]. As a deubiquitinating enzyme, OTUD1 facilitates ferroptosis by IREB2-mediated TRF1 expression in colon cancer cells, which in turn promotes the release of damage-associated molecular patterns (DAMPS) from dying cancer cells, subsequently, DAMPS enhances immune cell activity for killing to cancer cells [91]. As an eat-me signal, SAPE-OOH is distributed on ferroptotic cancer cells, which is recognized by TLR2 on macrophage leading to increased phagocytosis by macrophages [92], suggesting that ferroptosis induction promotes phagocytosis by macrophages. Therefore, ferroptosis modulates tumor immunotherapy by affecting immune cell activity including T cells and macrophages (Fig. 5).

## Future perspective

Ferroptosis is triggered by intracellular iron-mediated lipid ROS formation resulting in cell death. Conversely, SLC7A11/GPX4 axis protects cells from ferroptosis by detoxification. In this process, GPX4 exhibits antioxidant role in order to ferroptosis resistance, while it is unclear whether other GPX4 family members including GPX1-3 could inhibit ferroptosis. As an important ferroptosis regulator, ferritin undergoes autophagic degradation by NCOA4. Is there any other autophagy receptor for ferritin degradation? or does GPX4 undergo ubiquitination and degradation? Although SLC7A11 undergoes ubiquitination and degradation [57, 58], what is the specific ubiquitin ligase for SLC7A11 degradation? Does SLC7A11 undergo autophagic degradation? These issues need to be further determined. The mediators of ferroptotic cells could induce surround-cell ferroptosis [93], while the mechanism is still unclear. Although ferroptosis effectively enhances cancer immunotherapy [44, 83, 87], induction of ferroptosis could impair T cell survival [89]. Therefore, rational combined immune checkpoint inhibitors with ferroptosis inducers are essential for enhancement of cancer immunotherapy.
